# Equity assessment of childhood immunisation at national and subnational levels in Myanmar: a benefit incidence analysis

**DOI:** 10.1136/bmjgh-2021-007800

**Published:** 2022-07-08

**Authors:** Zin Mar Win, Tom Traill, Zarni Lynn Kyaw, Khaing Thandar Hnin, Phway Thinzar Chit, Thazin La, Ashwini Sunil Deshpande, Osondu Ogbuoji, Wenhui Mao

**Affiliations:** 1 Community Partners International, Yangon, Myanmar; 2 Department of Policy and Research, Community Partners International, Yangon, Myanmar; 3 Center for Policy Impact in Global Health, Duke Global Health Institute, Durham, North Carolina, USA

**Keywords:** Health economics, Health policy, Health services research, Immunisation

## Abstract

**Introduction:**

Myanmar, a conflict-affected geographically and ethnically diverse lower middle-income country, was in the donor transition phase for health prior to the political unrest of the last year. This study analyses the distribution of benefit and utilisation of basic childhood vaccinations from the highly donor-dependent Expanded Program on Immunization for populations of different socioeconomic status (SES).

**Methods:**

We conducted a benefit incidence analysis with decomposition analysis to assess the equity of benefit. We used basic childhood immunisations—BCG, measles, diphtheria, pertussis and tetanus (DPT)/pentavalent, oral polio vaccine (OPV) and full vaccination—as measurements for healthcare use. Childhood immunisation data were collected from Myanmar Demographic and Health Survey. Cost of vaccines was obtained from UNICEF document and ‘Immunization Delivery Cost Catalogue’ and adjusted with regional cost variations. We reported Concentration Index (CI) and Achievement Index (AI) by SES, including wealth quintiles, maternal education and across geographic areas.

**Results:**

Nationally, better-off households disproportionately used more services from the programme (CI–Wealth Index (CI-WI) for BCG, measles, DPT/pentavalent, OPV and full immunisation: 0.032, 0.051, 0.120, 0.091 and 0.137, respectively). Benefits had a pro-poor distribution for BCG but a less pro-rich distribution than utilisation for all other vaccines (CI-WI: −0.004, 0.019, 0.092, 0.045 and 0.106, respectively). Urban regions had a more pro-rich distribution of benefit than that in rural areas, where BCG and measles had a pro-poor distribution. Subnational analysis found significant heterogeneity: benefit was less equitably distributed, and AI was lower in conflict-affected states than in government-controlled areas. The major contributors to vaccine inequality were SES, antenatal care visits and paternal education.

**Conclusion:**

Donors, national government and regional government should better plan to maintain vaccine coverage while improving equity of vaccine services, especially for children of lower SES, mothers with less antenatal care visits and lower paternal education living in conflicted-affected remote regions.

**WHAT IS ALREADY KNOWN ON THIS TOPIC**

- Basic childhood immunisation coverage rates in Myanmar were lower than average rates for lower middle-income countries.
- Myanmar still has a high burden of vaccine preventable diseases.
- There was an inequality of childhood immunisation coverage across wealth quintiles by regions and states.

**WHAT THIS STUDY ADDS**

- Better-off households disproportionately used more services from the Expanded Program on Immunization (EPI) (Concentration Index–Wealth Index (CI-WI) for BCG, measles, DPT (diphtheria, pertussis and tetanus)/pentavalent, oral polio vaccine and full immunisation: 0.032, 0.051, 0.120, 0.091 and 0.137, respectively).
- Benefits had a pro-poor distribution for BCG but a less pro-rich distribution than utilisation (and lower coverage) for all other vaccines (CI-WI: −0.004, 0.019, 0.092, 0.045 and 0.106, respectively).
- Urban areas had a less equitable distribution of benefits than those rural areas.
- Subnational equity analysis found significant heterogeneity.
- In general, childhood immunisation inequity was more pronounced in urban poor and conflict-affected areas.
- Decomposition analysis revealed that wealth quintiles, number of antenatal care visits and paternal education contributed primarily to overall inequality in immunisation.
- Achievement Index, which considers both coverage and equity level, was the highest in the region of the second largest city, Mandalay, and the lowest in conflicted-affected state, Rakhine.

**HOW THIS STUDY MIGHT AFFECT RESEARCH, PRACTICE AND/OR POLICY**

- The findings of this study should inform national and subnational decision-makers and donor agencies in their future planning of childhood vaccination and donor transition.
- Poor and less educated populations, children of mothers with less number of antenatal care visits especially in urban and conflicted-affected areas, need more attention to improve the service uptake of EPI and improve equity.

## Introduction

The 2010 World Health Report identified inequitable use of healthcare resources as one of the main barriers to achieving universal health coverage (UHC).^1^ In most countries, the poor tend to have more healthcare needs but receive less healthcare.^2 3^ Therefore, strategies to promote equity in resource distribution are expected to improve the use of healthcare services relative to need and financial protection, the targets of UHC.^4^

Immunisation is widely recognised as one of the world’s most successful global health programmes in terms of reaching poor populations. Myanmar’s Expanded Program on Immunization (EPI) was launched in 1978, starting in 104 townships, and reaching almost all townships by 1997.^5^ BCG, diphtheria, pertussis and tetanus (DPT) and tetanus toxoid (TT) were first introduced in 1978 and measles and oral polio virus (OPV) were subsequently added into the routine EPI in 1987.^5^ Since 2012, DPT and hepatitis B have been combined with *Haemophilus influenza* type b as pentavalent vaccine.

One of the EPI’s objectives is to improve access to immunisation services and to provide equitable service to all target populations.^6^ However, immunisation coverage rates in Myanmar are lower than the averages rates for lower middle-income countries (LMICs). In 2015, the coverage rate for BCG, measles, 3 doses of DPT/pentavalent and OPV were 88%, 77%, 62% and 55%, respectively, in Myanmar, compared with 85%, 81%, 80% and 81%, respectively, on average across all LMICs.^7^ Myanmar still has a high burden of vaccine-preventable diseases. In 2018, 1389 cases of measles, 127 cases of diphtheria, 28 cases of pertussis and 22 cases of neonatal tetanus were reported. In addition, 4 cases of circulating vaccine-derived poliovirus type 1 were detected in 2019.^8 9^ Immunisation coverage can be used as a proxy for monitoring overall healthcare performance.^10^ Encouraging equity for immunisation services will not only make achieving global immunisation coverage targets possible but may also improve coverage of other primary healthcare interventions.^5 6^

The geographic and ethnic diversity of Myanmar adds more complexity to achieving health equity. The nation is composed of eight regions and seven states. One of the regions, Naypyitaw, where the capital city is located, is known as the union territory. All regions and states (R&S) are constitutionally equivalent. The majority ethnic group, Bamar (70% of the whole population), constitutes the majority across the country’s eight regions, namely, Ayeyarwady, Bago, Magway, Mandalay, Naypyitaw, Sagaing, Tanintharyi and Yangon.^11 12^ The seven states named after the seven main minority ethnic groups—Kachin, Kayah, Kayin, Chin, Mon, Rakhine and Shan—have seen the bulk of the conflict since Myanmar’s independence in 1948. In Mon State, there remains contestation (and some areas are out of government control) but not fighting at present. In Chin, Kayin, Kayah, Kachin, Tanintharyi and Shan states, conflict remains active preventing the equitable distribution of health services in the states for ethnic minority populations (while Rakhine State saw some of the most intense fighting in the last 5 years until 2020). Additionally, accessibility for the government, language barriers and trust are also barriers to health services. In general, while regions are located in the central part of the country (except Tanintharyi region) residing higher population, states are located along country’s borders residing fewer population.^12–14^ Background information on these regions and states is in online supplemental table S1.

10.1136/bmjgh-2021-007800.supp1 Supplementary data

Myanmar became an LMIC in 2015 and donors will be withdrawing their external aid in the coming years. According to the Myanmar National Health Accounts, external financing made up more than 50% of total expenditure for preventive care such as immunisation programmes, epidemiological surveillance and risk and disease control programmes.^15^ The government spent around 5% of gross domestic product on healthcare over 2015–2017. In 2015, 2016 and 2017, domestic general government health expenditure as a percentage of current health expenditure (CHE) was 22%, 14% and 15%, respectively, and out of pocket payments were 70%, 77% and 76% of CHE, respectively. High dependency on external financing for some services, low government health spending and high private health spending remain the biggest obstacles in the sustainable financing of health after transition.

In Myanmar, basic childhood immunisation is one of the rare programmes with no de facto user fees and has been widely used by Myanmar population, including those better-off families.^16^ Yet, the EPI is also one of the health programmes that relies heaviest on international development assistance. Gavi, the Vaccine Alliance, was the largest contributor to Myanmar’s immunisation programme in 2015, contributing 56% of total finance for the programme. The government contributed 7% and the remainder was financed by UNICEF, WHO, the Three Millennium Development Goals Fund and other development partners.^6^ Total immunisation expenditure was approximately US$58 million divided as follows: US$30 million for routine immunisation and US$28 million for supplementary immunisation activities.^6 17^ If financing cannot be adequately shifted to domestic resources, donor transition will have a negative impact on the heavily donor-dependent immunisation programme and health service use by households in Myanmar.

Previous studies have analysed the distribution of benefits from public healthcare services in India, Bangladesh, Cambodia and Nigeria.^18–21^ To the best of our knowledge, no studies have as yet assessed the benefit of government-funded and donor-funded childhood immunisation in Myanmar. This study aims to analyse the distribution of benefit from donor-funded and government-funded childhood vaccination programmes for populations of different socioeconomic status (SES) and across geographic areas.

## Methods

### Benefit incidence analysis

Benefit incidence analysis (BIA) describes the distribution of benefits in monetary terms, and theoretically, it can be calculated by multiplying the healthcare utilisation rate by the unit cost of the intervention that can vary across different healthcare settings or geographical areas, and then subtracting any payments borne by the individual.^4 22 23^

In this study, we assessed the distribution of benefits from public and donor financed immunisation programmes for different childhood vaccines across different SES at both national and subnational levels. The following components were involved in our study:

1. Utilisation: childhood immunisation uptake data across wealth quintiles and maternal education levels for each R&S, and urban and rural areas were collected.
2. Unit cost: the total unit costs per dose for each vaccine were calculated using the unit cost per dose of different types of vaccines and adding estimated delivery costs per dose that varied across different R&S and urban and rural areas. As the basic immunisation programme was heavily funded by donors and had no user fee, we did not consider private spending in this study.
3. Benefits: the monetary value of each immunisation benefit to the population groups of interest was then calculated by multiplying the immunisation uptake rates and unit costs per dose of vaccination.

### Data collection

#### Population data

Secondary data related to childhood immunisation uptake was collected from the nationally representative Myanmar Demographic and Health Survey (MDHS). This survey was the first Myanmar DHS conducted from December 2015 to July 2016, as part of the International DHS programmes. Details of sampling and weighing methods can be seen in the MDHS report 2015–2016.^24^

We specifically extracted the following immunisation uptake information among 12–23-month-old children in Myanmar: one dose of BCG, one dose of measles, three doses of DPT/pentavalent and three doses of OPV. Then, we analysed full immunisation, which refers to 12–23-month-old children who have received 1 dose each for BCG and measles and 3 doses each for DPT/pentavalent and OPV.^25^ Vaccination status was assessed in two ways: a record on the child’s vaccination card and mother’s recall when there was no card. To examine the contribution of each variable to vaccine inequality, the following variables were selected: wealth quintiles, urban/rural areas, conflict areas, mother’s education, child’s gender, number of antenatal care visits, place of delivery, father’s education, marital status, mother’s age and maternal tetanus immunisation status.^26^

Household Wealth Index (WI) scores were calculated using principal component analysis based on the household ownership of goods and housing characteristics.^24 27^ Each household member living in the same household was assigned same household WI scores, ranked and divided into 5 equal-sized wealth quintiles (each with 20% the population). Mother’s education levels (ME)—no education, primary, secondary and more than secondary—were classified according to the UNESCO’s International Standard Classification of Education.^27^

#### Unit cost

Unit costs for the different types of vaccines and syringes were obtained from ‘costs of vaccinating a child’ published by UNICEF.^28^ Vaccine delivery cost was obtained from the ‘Immunization Delivery Cost Catalogue’ (IDCC), which was calculated to estimate immunisation delivery unit cost for low-income and middle-income countries. This IDCC was developed by the Immunization Costing Action Network, led by ThinkWell and John Snow.^29^ To consider geographical variations in vaccine delivery cost, average vaccine delivery cost obtained from IDCC was adjusted. Aye *et al* reported the operational costs of routine immunisation activities at urban and rural subcentres of different geographic areas: hilly plateau area, delta area, central plain area, coastal area and mountain range area.^30^ The average operational costs in rural areas were found out to be higher than urban areas in all geographic regions except the hilly plateaus area—Shan State—where immunisation operational costs in urban area were slightly higher than rural area and central plain areas—Mandalay, Yangon, Naypyitaw, Sagaing and Magway—where immunisation operational costs in urban and rural were the same. We calculated percentage increase and decrease from average operational costs for different geographic areas and applied it to average vaccine delivery cost accordingly. All the costs in this study are reported in 2016 in US$.

The vaccine cost, injection supply cost and delivery costs by R&S can be found in online supplemental table S2. A list of variables and data sources can be seen in online supplemental table S3.

### Data analysis

#### Concentration curve

We used StataCorp 2015 and Microsoft Excel for the data analysis. Concentration curves (CCs) were used to describe the health benefits and utilisation incidence by plotting the cumulative proportion of children in different wealth quintiles on the x-axis against cumulative proportion of health benefits (number of immunisations across SES multiplied by unit cost) and utilisation (number of immunisations across SES) on the y-axis, respectively. If a CC lies above the 45° line, the benefits disproportionately go to the lower wealth quintiles and have a pro-poor distribution. Conversely, if the CC lies below the 45° line, the rich receive more health benefits with a pro-rich distribution.

#### Concentration index

The Concentration Index (CI) measures relative equity and is defined as twice the area between the CC and the 45° equity line. The value of the CI ranges from −1 to +1. A negative value indicates that health service utilisation/benefits is concentrated among lower SES and a positive value indicates service utilisation/benefits concentrated among higher SES.^22 31^

Decomposition analysis (DA) of CI quantifies the extent to which individual variables contribute to overall inequality. It is estimated using a regression-based approach: generalised linear model for vaccine utilisation and ordinary least square for vaccine benefits.^22 32^

#### Achievement Index

The Achievement Index (AI), developed by Wagstaff, reflects both the average level of health service utilisation and the inequality of the distribution.^33 34^

AI(v) = µ(1−C(v)).

The average coverage of a healthcare intervention, µ, was multiplied by one minus the CI, C(v), to obtain the AI, AI(v). The inequality aversion (IA) parameter, v, captures the extent of societal aversion to health inequality. When v=2, C(2) is equal to standard CI. When v>2, the weight attached to the health of lower SES increases, while the weight attached to the health of higher SES falls. Therefore, aversion to socioeconomic inequalities in health increases. When applied to positive health measures like immunisation rates, a higher value of the AI represents greater achievement. Higher achievement can be obtained either by improving average coverage or by reducing inequality.

## Results

Among the four types of vaccines (BCG, measles, DPT/pentavalent and OPV), coverage was highest for BCG (87.8%) and lowest for three doses of DPT/pentavalent (62.3%). More than half of children (54.8%) received full immunisation, of which urban area had more coverage (67.47%) than rural area (50.40%). Subnationally, vaccine coverage varied significantly. Mandalay Region (81.3%) had the highest coverage for full immunisation, whereas the lowest was found in Ayeyarwady (33.8%) (online supplemental table S5). The percentage and number of 12–23-month-old children sample living across five different wealth quintiles according to regions/states and urban/rural areas can be seen in online supplemental table S4. Immunisation coverage according to WI and ME can be found in online supplemental tables S6 and S7.

### Utilisation and benefit incidence across wealth quintiles

**Figure 1:**
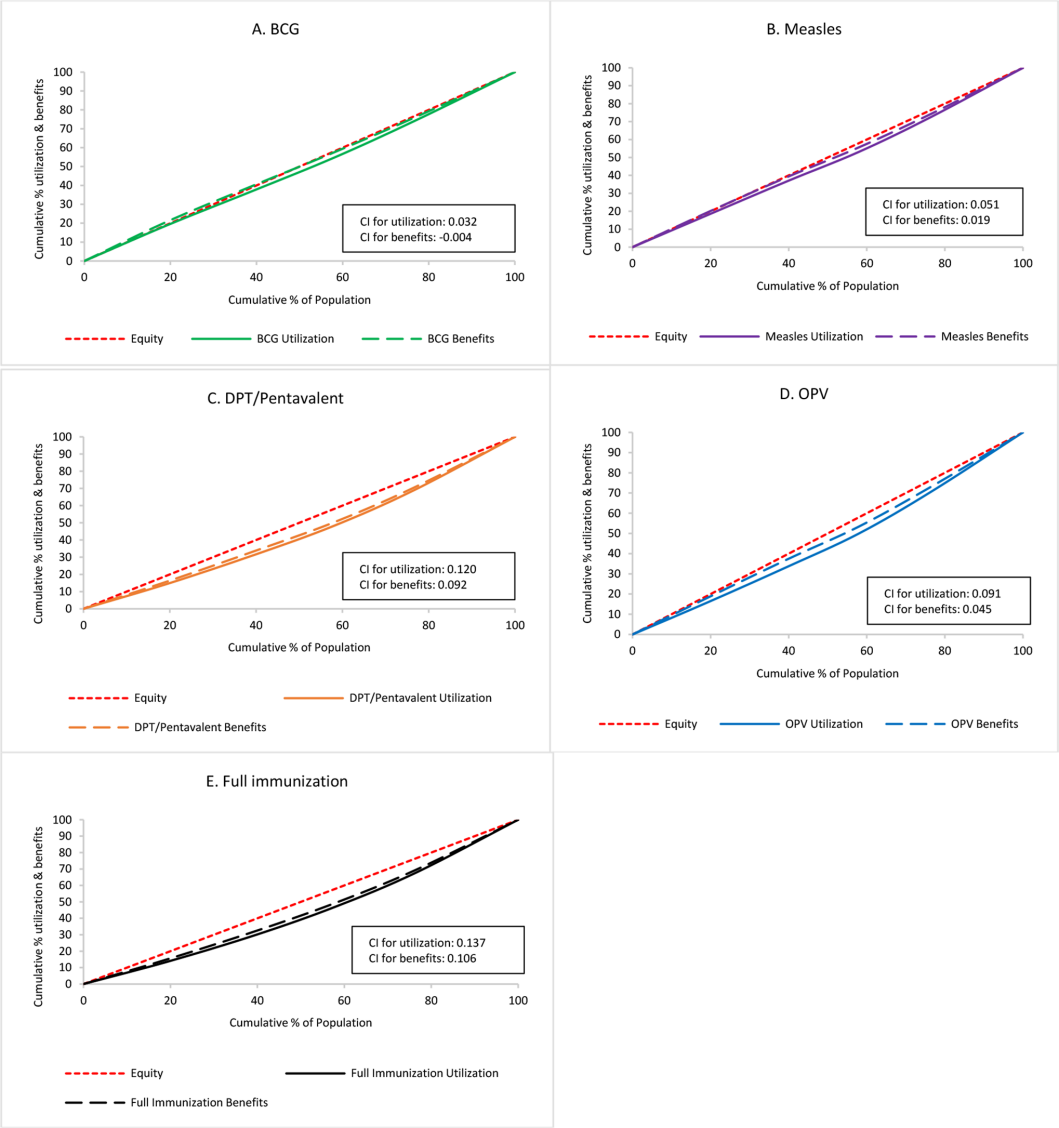
National level immunisation utilisation and benefits across wealth quintiles for (A) BCG, (B) measles, (C) DPT/pentavalent, (D) OPV and (E) full immunisation. CC, concentration curve; CI, Concentration Index; DPT, diphtheria, pertussis and tetanus; OPV, oral polio vaccine.

National level comparison of immunisation utilisation and benefit incidence across wealth quintiles depicted in CCs (CCs for utilisation (CC-WI-U) vs CCs for benefits (CC-WI-B)) and CIs (CIs for utilisation (CI-WI-U) vs concentration indices for benefits (CI-WI-B)) is shown in figure 1. BCG was distributed most equitably (pro-poor), while measles, OPV and DPT/pentavalent all showed pro-rich distributions. Full immunisation had the most unequal distribution. CC and CI in figure 1 reveal that benefit distribution was generally less inequitable than utilisation. BCG had a pro-rich pattern (CI-WI-U: 0.032) in utilisation distribution but had a pro-poor pattern (CI-WI-B: −0.004) in benefit distribution. All other immunisations, which were pro-rich in utilisation, were less in benefit distribution (CI-WI-U vs CI-WI-B for measles, DPT/pentavalent, OPV and full immunisation: 0.051 vs 0.019, 0.120 vs 0.092, 0.091 vs 0.045 and 0.137 vs 0.106, respectively).

**Figure 2:**
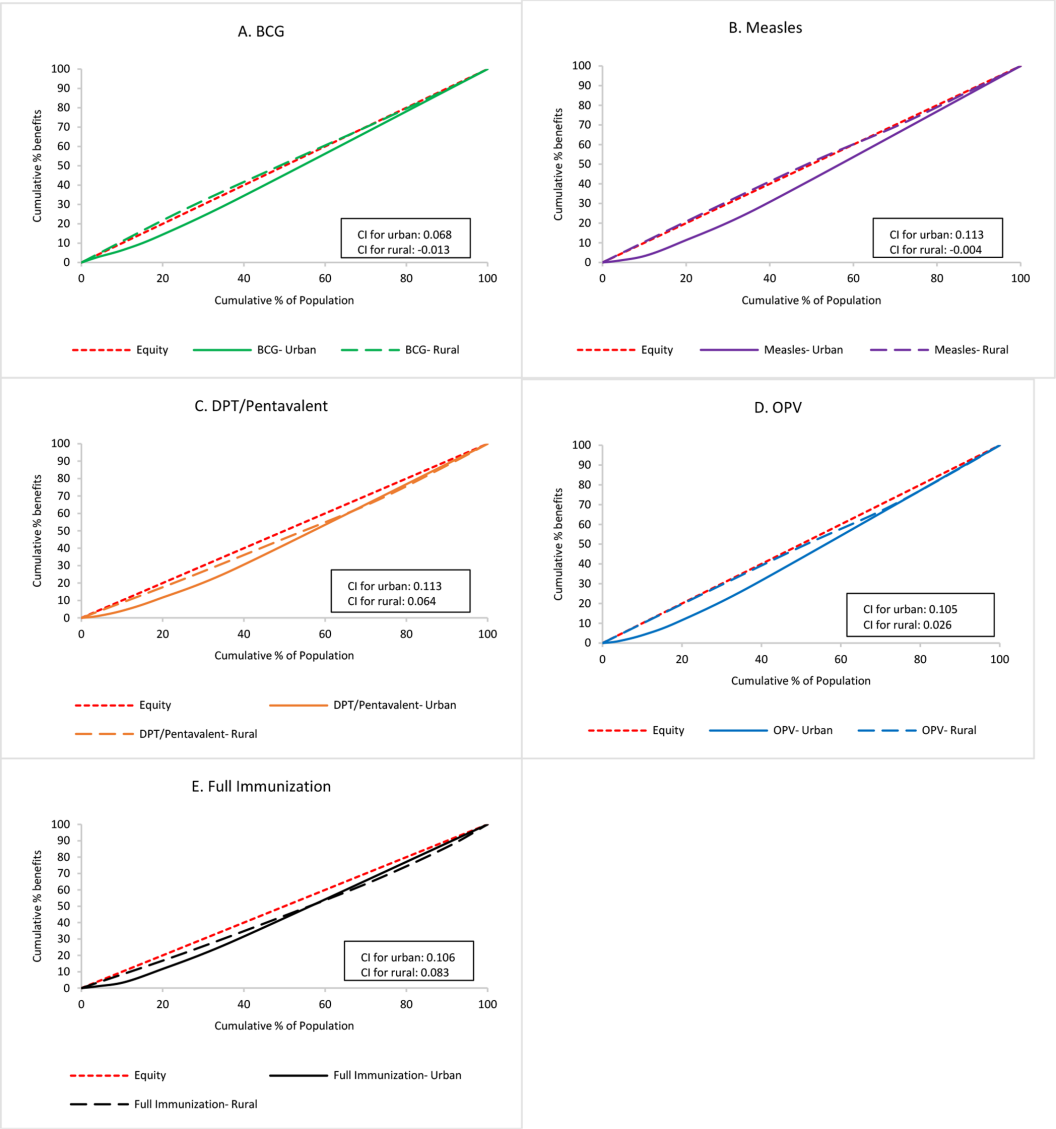
Comparison of urban/rural immunisation benefits across wealth quintiles for (A) BCG, (B) measles, (C) DPT/Pentavalent, (D) OPV and (E) full immunisation. CC, concentration curve; CI, Concentration Index; DPT, diphtheria, pertussis and tetanus; OPV, oral polio vaccine.

Immunisation benefits across wealth quintiles stratified by urban and rural population can be seen in figure 2. It is quite interesting that although immunisation coverage was higher in urban areas, benefit incidence was more equal in rural areas for all vaccines. The immunisation benefit difference between urban and rural areas was relatively higher for BCG, measles and OPV (CI-WI-B for urban vs rural areas: 0.068 vs −0.013, 0.113 vs −0.004 and 0.105 vs 0.026, respectively) and less so for DPT/pentavalent and full immunisation (CI-WI-B for urban vs rural: 0.113 vs 0.064 and 0.106 vs 0.083, respectively). CIs in table 1 shows that benefit incidence was more equal than utilisation incidence in rural areas, but it was not in urban areas.

**Table 1:** CIs of different types of immunisations by wealth quintiles at rural and urban areas and subnational levels (CI-WI-U and CI-WI-B)

	BCG	Measles	DPT	OPV	Full immunisation
**Urban**					
CI for utilisation	0.059	0.106	0.109	0.099	0.102
CI for benefits	0.068	0.113	0.113	0.105	0.106
**Rural**					
CI for utilisation	0.013	0.017	0.085	0.062	0.106
CI for benefits	−0.013	−0.004	0.064	0.026	0.083
**Mandalay**					
CI for utilisation	0.003	0.007	−0.012	0.000	−0.009
CI for benefits	0.003	0.007	−0.012	0.000	−0.009
**Kayah**					
CI for utilisation	0.000	0.015	0.021	0.049	0.070
CI for benefits	−0.014	0.003	0.011	0.036	0.060
**Yangon**					
CI for utilisation	0.019	0.099	0.087	0.096	0.115
CI for benefits	0.019	0.099	0.087	0.096	0.115
**Sagaing**					
CI for utilisation	−0.006	−0.026	0.032	0.032	0.018
CI for benefits	−0.006	−0.026	0.032	0.032	0.018
**Kayin**					
CI for utilisation	0.040	0.031	0.093	0.045	0.111
CI for benefits	0.029	0.023	0.083	0.030	0.100
**Mon**					
CI for utilisation	0.023	0.068	0.107	0.080	0.120
CI for benefits	−0.036	0.017	0.061	0.021	0.065
**Kachin**					
CI for utilisation	0.012	0.039	0.099	0.089	0.120
CI for benefits	−0.012	0.022	0.081	0.061	0.099
**Magway**					
CI for utilisation	0.008	0.009	0.014	0.097	0.060
CI for benefits	0.008	0.009	0.014	0.097	0.060
**Chin**					
CI for utilisation	0.029	0.108	0.088	0.054	0.075
CI for benefits	0.016	0.097	0.082	0.044	0.069
**Tanintharyi**					
CI for utilisation	0.016	0.046	0.131	0.098	0.156
CI for benefits	−0.003	0.029	0.113	0.075	0.133
**Naypyitaw**					
CI for utilisation	−0.004	0.021	0.182	0.261	0.308
CI for benefits	−0.004	0.021	0.182	0.261	0.308
**Bago**					
CI for utilisation	−0.022	−0.012	0.071	0.083	0.112
CI for benefits	−0.052	−0.039	0.042	0.047	0.079
**Shan**					
CI for utilisation	0.139	0.154	0.233	0.210	0.211
CI for benefits	0.140	0.155	0.233	0.210	0.212
**Rakhine**					
CI for utilisation	0.008	0.105	0.164	0.075	0.201
CI for benefits	−0.012	0.083	0.141	0.045	0.173
**Ayeyarwady**					
CI for utilisation	−0.043	−0.012	−0.115	−0.117	−0.104
CI for benefits	−0.063	−0.040	−0.142	−0.144	−0.126

B, Benefits; CI, Concentration Index; DPT, diphtheria, pertussis and tetanus; OPV, oral polio vaccine; U, Utilisation; WI, Wealth Index.

Subnational level comparison of CI-WI for utilisation and benefit distributions can be found in table 1. Among 15 R&S, immunisation distribution was most equal in Ayeyarwady Region and Mandalay Region and most unequal in Naypyitaw Region, Shan and Rakhine States. CIs revealed that benefits were more equally distributed than utilisation in Kayah, Kayin, Mon, Kachin, Chin, Tanintharyi, Bago, Rakhine and Ayeyarwady, where immunisation delivery cost was also higher in rural than urban areas. In contrast, benefit distribution was slightly more unequal than utilisation in Shan State where urban immunisation delivery costed slightly more than rural.

Decomposition of CI for full immunisation utilisation and benefits can be seen in online supplemental table S8. The major contributors that increase the inequality of vaccine utilisation and benefits were SES, antenatal care visits and paternal education.

Estimated monetary benefits received per quintile for each vaccination can be seen in table 2. The number of 12–23-month-old children living in each wealth quintile was divided into R&S and urban and rural areas depending on where they came from and multiply it with different vaccination cost for different R&S and urban/rural area accordingly. The richest children received a higher coverage and a higher share of estimated monetary benefits relative to their population for all vaccine types. Although the coverage for the poorest children was not that high, share of estimated monetary benefits for BCG and measles seemed to be equal.

**Table 2:** Estimated monetary benefits per quintile for each vaccination

	Poorest	Poorer	Middle	Richer	Richest	Total^*^
Number of children^†^	176 652 (19.54%)	183 774 (20.33%)	164 318 (18.18%)	167 248 (18.50%)	212 008 (23.45%)	904 000
BCG uptake (%)	86.36	80.15	81.94	91.15	97.51	
Estimated benefits for BCG	405 171 (21.16%)	370 406 (19.34%)	326 311 (17.04%)	348 878 (18.22%)	464 033 (24.23%)	1 914 798
Measles uptake (%)	71.67	71.22	68.77	81.17	89.83	
Estimated benefits for measles	409 479 (19.71%)	407 041 (19.60%)	340 377 (16.39%)	388 336 (18.69%)	531 991 (25.61%)	2 077 225
DPT/pentavalent uptake (%)	46.53	52.07	57.15	69.57	82.58	
Estimated benefits for DPT/pentavalent	801 234 (15.99%)	890 854 (17.78%)	828 188 (16.53%)	1 009 923 (20.15%)	1 480 992 (29.55%)	5 011 191
OPV uptake (%)	55.37	57.81	60.32	74.98	83.73	
Estimated benefits for OPV	725 292 (18.47%)	742 146 (18.90%)	632 446 (16.10%)	774 161 (19.71%)	1 053 544 (26.82%)	3927 589
Full immunisation uptake (%)	38.60	44.02	51.42	61.37	75.17	
Estimated benefits for full immunisation	1 551 472 (15.27%)	1 747 136 (17.19%)	1 726 887 (16.99%)	2 045 258 (20.13%)	3 091 645 (30.42%)	10 162 398

*Estimate monetary benefits are shown in 2016 USD.

†According to United Nations Population Division, the number of 2-year-old children that lived in Myanmar in 2016 were estimated to be 904 000.^41^

DPT, diphtheria, pertussis and tetanus; OPV, oral polio vaccine.

### Utilisation and benefit incidence across maternal education

National level comparison of immunisation utilisation and benefit incidence by maternal education illustrated in CCs (CC-ME-U vs CC-ME-B) and CIs (CI-ME-U vs CI-ME-B) are shown in online supplemental figure S1A. Like the distribution across wealth quintiles, CC and CI in online supplemental figure S1A showed that all immunisations are proeducated in utilisation, but less pro-educated in benefits (CI-ME-U vs CI-ME-B for BCG, measles, DPT/pentavalent, OPV and full immunisation: 0.034 vs 0.011, 0.037 vs 0.017, 0.082 vs 0.063, 0.061 vs 0.034 and 0.073 vs 0.051, respectively). Among all immunisations, BCG was distributed the least unequally, followed by measles, OPV, full immunisation and DPT/pentavalent, which had the most unequal distribution.

10.1136/bmjgh-2021-007800.supp3 Supplementary data

Immunisation benefits across maternal education stratified by urban and rural population can be seen in online supplemental figure S1B. Like the urban and rural analysis for wealth quintiles, benefit incidence was more equal in rural populations than urban populations (CI-ME-B for BCG, measles, DPT/pentavalent, OPV and full immunisation in urban area vs rural area: 0.037 vs 0.015, 0.062 vs 0.011, 0.075 vs 0.043, 0.067 vs 0.026 and 0.079 vs 0.022, respectively). CIs in online supplemental table S9 showed that benefit distribution was slightly more equal than utilisation in both rural area and urban area.

Subnational level comparison of CI-ME for utilisation and benefit distributions can be found online supplemental table S9. At R&S level, immunisation benefits across mother’s education were distributed most equally in Ayeyarwady and Mandalay Regions and were distributed most unequally in Naypyitaw Region, Rakhine and Shan States. Similarities and differences between benefit and utilisation incidence across maternal education by different R&S were the same as that across wealth quintiles. For example, benefit was more equally distributed than utilisation in R&S where immunisation delivery cost was higher in rural than urban area.

### Achievement Index

AI is composed of both vaccine coverage and distribution of benefits. Mandalay Region had the highest AI (ranked 1 in both AI-WI and AI-ME); this region had the highest vaccine coverage and equity was the second highest (online supplemental table S10). In contrast, Rakhine State had the lowest AI (ranked 15 in both AI-WI and AI-ME) with the second lowest coverage, third lowest equity by wealth quintiles and second lowest equity by maternal education. It is noteworthy that although Ayeyarwady had the lowest coverage (ranked 15), it was rewarded by highest equity (ranked 1) and its achievement was ranked 12 by WI and 14 by ME. Rakhine State, ranked 14 by coverage, was penalised by a low level of equity and its achievement was ranked the lowest, 15 by both WI and ME.

**Figure 3:**
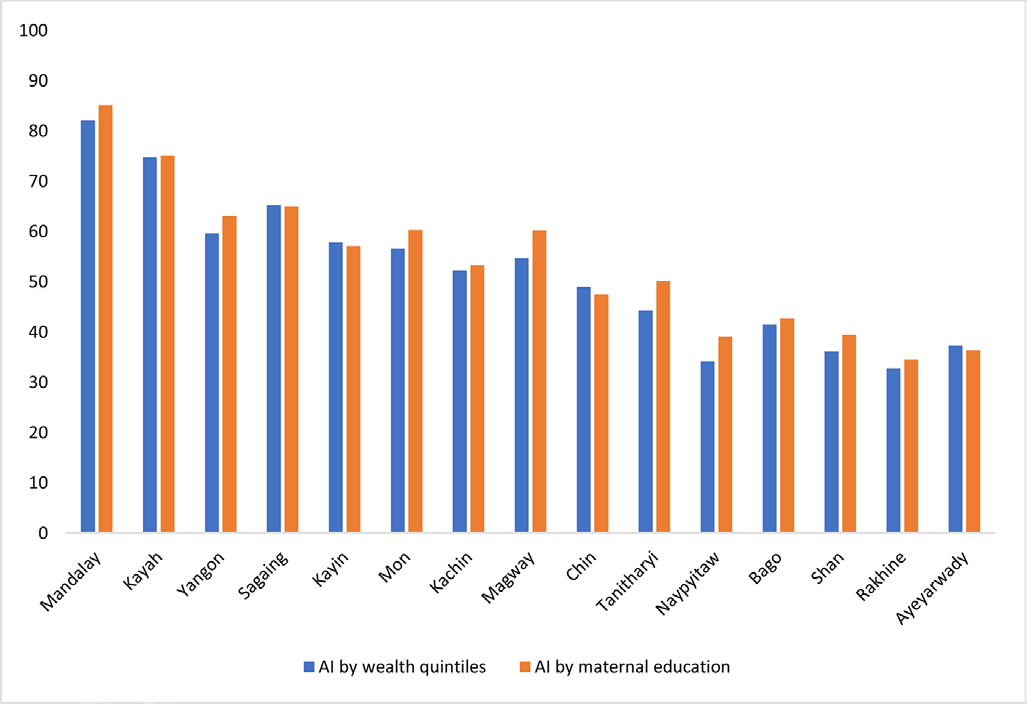
I by wealth quintiles and maternal education across R&S. AI, Achievement Index; R&S, regions and states.

Nationally, AI-WI (47.29) was lower compared with AI-ME (50.82). That means distribution of benefit was less equitable across wealth quintiles than mother’s education levels. Figure 3 compares AI-WI and AI-ME at subnational level. All R&S except Sagaing, Kayin, Chin and Ayeyarwady have higher AI across maternal education than that of wealth quintiles.

**Figure 4:**
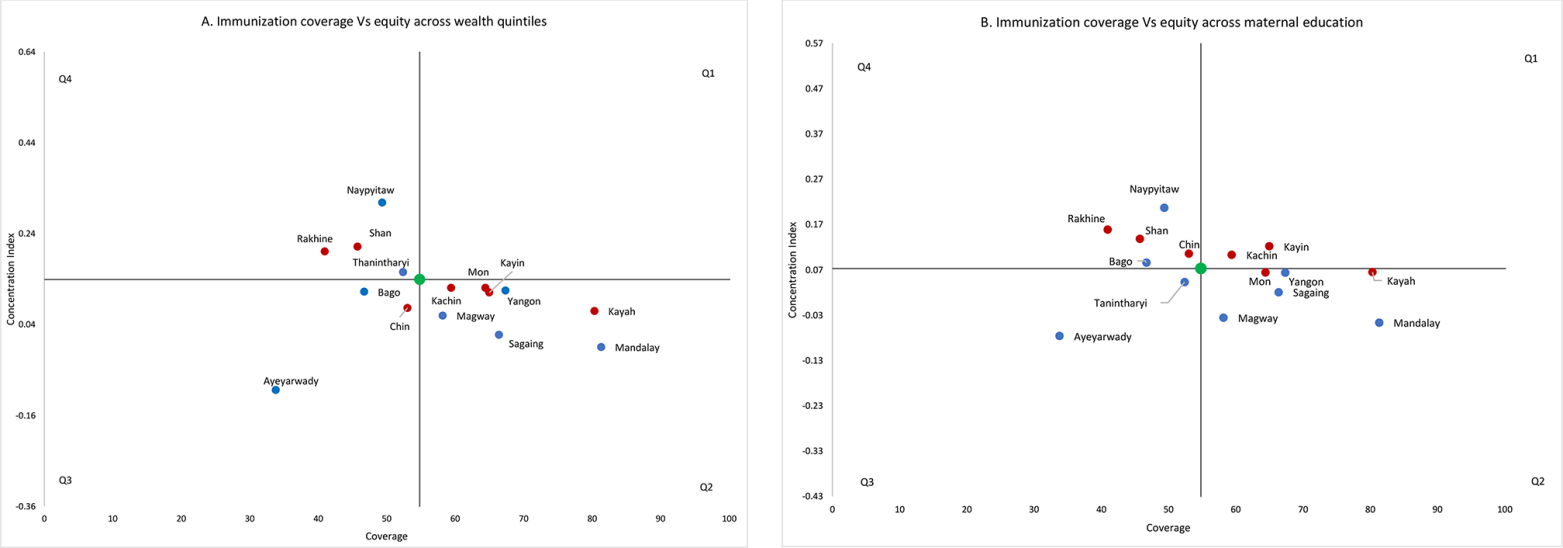
National and subnational level immunisation coverage and CIs by (A) wealth quintiles and (B) maternal education. CIs, Concentration Indices.

Four quadrant planes were plotted using immunisation coverage on x-axis and CI-WI and CI-ME on y-axis (figure 4). National average indicators being at the centre, R&S seen in Q2 demonstrates better coverage and more equitable distribution, Q4 worse coverage and less equitable distribution, Q1 better coverage but less equitable distribution and Q3 worse coverage but better equity than national coverage.

### Sensitivity analysis

We performed sensitivity analysis by changing IA parameters to see how our measure of inequality varies when the weight attached to the health of worst-off population changes. Generally, raising the value of AI parameter, v, above 2 results in pronounced pro-rich inequality. We applied v=4 and v=6 in both CI-WI and CI-ME, and also measured their related AI (online supplemental table S11 and S12).

The impact of raising IA varies across R&S. When v value was raised from 2 to 6, CI-WI increased more than threefolds from 0.070 to 0.239 in Kayah and from 0.060 to 0.214 in Magway, implying immunisation coverage among the poorest quintile is much lower than that among the other four quintiles. In contrast, in Kayin, the change was quite small, from 0.110 to 0.131, suggesting that the immunisation coverage rate in the poorest quintile is similar to that of the second poorest quintile. When v value was raised from 2 to 6, CI-MEI increased nearly threefold, from 0.065 to 0.191, in Kayah because coverage in the lowest maternal education group differed dramatically from the rest of the groups. In contrast, only minor change was observed in Tanintharyi, from 0.043 to 0.068, which reflects the fact that the coverage rate in the lowest education group is not much different from that in the second lowest education group. Based on the extent CI changed, AI varied accordingly.

Although changes in CI-WI and CI-ME in Naypyitaw and Shan had not reached three times on our choice of IA parameters, their absolute changes were quite significant, reflecting that the poorest quintile suffered more than among the other four quintiles. For example, when v value was raised from 2 to 6, CI-WI increased from 0.308 to 0.712 and from 0.211 to 0.461 in Naypyitaw and Shan, respectively. Similarly, CI-ME increased from 0.207 to 0.429 and from 0.138 to 0.311 in Naypyitaw and Shan, respectively.

## Discussion

This is the first study that reported benefit and utilisation distribution across SES at national, urban, rural and subnational levels from the highly donor-funded EPI and estimated the population groups that could potentially be affected more from donor transition. We used CC, CI and AI to assess both vaccine coverage and the distribution of benefits and utilisation, and DA to explore key contributors of inequality in full immunisation. We also conducted sensitivity analysis to test our findings. We observed many interesting and useful findings. At national level, better-off households disproportionately used more services from the programme and showed pro-rich distributions. Benefit had a less pro-rich distribution than utilisation did. BCG was the most equitable vaccine, showing a pro-poor distribution of benefit incidence.

The more equitable distribution of benefits than utilisation at national level captures the greater vaccine delivery cost for poorer parts of the country. Households of lower SES tend to live in rural areas of remote R&S; the higher vaccine transportation and travel costs required for service delivery mean that the cost to serve these communities is greater, so benefit distribution is more equitable. Nonetheless, more money was spent delivering vaccines to the richest children, who received a higher coverage and as a result enjoyed a greater share of the benefits relative to their population size for all vaccines. Although coverage for the poorest children was not as high, for BCG and measles, the benefit was close to fair because of the high vaccine transportation costs in rural and remote areas as mentioned above. Our analysis showed higher vaccine coverage tends to be associated with more equitable distribution in general, which contributed to higher AI.

A comparison within rural and urban areas showed that benefit incidence was more equal than utilisation incidence in rural areas, but the difference was not significant in urban areas. This was driven by variation in costs for rural areas, with some remote R&S where many of rural poor reside, costing significantly more than in central R&S (full immunisation cost in rural remote R&S like Tanintharyi, Rakhine and Mon was US$24.92 vs in rural central R&S like Mandalay, Yangon and Naypyitaw was US$18.92), while urban delivery costs were broadly similar. Comparing between urban and rural areas, benefit incidence was more equal in rural areas for all vaccines. This was due to greater equality in utilisation and higher vaccine delivery costs in rural areas of remote R&S than that in urban areas. Urban poor received the less benefits because of greater inequity in utilisation and lower vaccine delivery costs in urban areas of different R&S than that in rural areas.

Like urban and rural analysis, the difference between utilisation and benefit at subnational level tended to be associated with variations of vaccine delivery cost difference among urban and rural area of each R&S. If vaccine delivery cost was higher in rural than urban, the benefit distribution was more equitable than utilisation in that region or state (eg, Kayah, Tanintharyi, Rakhine, etc). In contrast, if vaccine delivery cost was higher in urban area than rural area, the benefit distribution was less equitable than utilisation in that region or state (eg, Shan State).

Subnational equity analysis showed significant heterogeneity. Ayeyarwady and Mandalay had the most equitable distributions. In contrast, Naypyitaw, Shan and Rakhine had the least equitable distribution of benefits from EPI. In general, we found economic development appeared to be less directly linked to equity of vaccine delivery, but conflicted-affected areas appeared to have both poor vaccine coverage and less equitable distributions of benefits. These can be seen through a few examples. The two most equitable R&S are Ayeyarwady and Mandalay, two peaceful regions at opposite ends of economic development. Ayeyarwady (delta) is a relatively resource poor area that has fewer health facilities than the national average and in which people earn lower average income per capita. Even though it has low vaccine coverage, the distribution of benefit is pro-poor. Mandalay is the second largest city with better economic development and more healthcare facilities. Mandalay had good performance in both vaccine coverage and equitable distribution. In contrast, Shan State and Rakhine State have seen ongoing, complex conflicts in the last decade. Rakhine has the lowest income per capita in Myanmar. Both states had poor performance in both vaccine coverage and inequitable distribution.

The results of DA identified underlying factors contributed to the vaccine inequality and policy-makers and donors should prioritise children of lower SES population, mothers with less antenatal care visits and lower paternal education to improve vaccine equality for children in Myanmar.

In previous study for Myanmar, Han *et al* assessed quintile-specific inequalities in access to health services in Myanmar by using the slope index of inequality.^35^ They reported that there were notable differences in inequality of full immunisation coverage across all R&S. Our study echoes the findings of Grundy and Biggs, who pointed out that central urban areas like Mandalay had the highest coverage rate and Rakhine State, where intercommunal violence has occurred over the last couple years, had the second lowest coverage.^36^ Our analysis added further valuable results revealing that Mandalay was the second most equitable and Rakhine was the one of the least equitable areas in the distribution of EPI benefit.

Our study has several limitations. First, we extracted the immunisation uptake information from the national MDHS dataset using both the child’s vaccination card and mothers’ recall and this raises the question of the validity of mother’s recall. Several previous studies suggested the high validity of recall-based data, while others have reported errors in mothers’ recall.^37–40^ Second, we used cost information from ‘Assessing the operational cost of routine immunisation activities at the subcentre level in Myanmar: what matters for increasing national immunisation coverage?’ and estimate the cost by region and by urban/rural area. There might be variance among different facilities, with some areas in conflict-affected parts of the country reliant on vaccination campaigns having significantly higher costs. Third, BIA only assesses quantitative data like the number of vaccinations and unit cost, and quality of services is not considered. Finally, although DHS is a nationally representative households survey, the sample size for 12–23-month-old children in each 15 R&S was relatively small and this results in limited statistical power.

Despite such limitations, this study provides valuable evidence to inform national and subnational decision-makers and donor agencies in their future planning of childhood vaccination and donor transition. The Myanmar EPI has been financed mainly by Gavi, the Vaccine Alliance, along with other development partners and a small share of contributions from government. Gavi and other donors paid for vaccines and supplies, cold chain equipment, training, microplanning, etc. The government’s fund was used mainly for human resources, the facilities and operational costs. As Myanmar is now classified as LMIC, it has entered the preparatory transition phase. In 2017, government started taking responsibility for the cost of traditional vaccines together with its share of co-financing for new vaccines such as pentavalent, pneumococcal and Japanese encephalitis etc.

Myanmar was projected to enter accelerated transition phase in 2021, where co-financing responsibilities would increase significantly. After the coup and the significant economic contract of the last 2 years, however, this may be delayed. Although Myanmar has some co-financing arrangements with Gavi, the Vaccine Alliance, and has fulfilled its financing obligations for vaccine costs, there is significant uncertainty about its future funding commitments to the EPI.^17^ In the short term, in order to continue delivering vaccines, it is necessary for donors to work with multiple partners to deliver to mothers and children in Myanmar, while the government should also prioritise and increase the government’s health budget.

As and when donors, including Gavi, do leave the country, this study provides valuable recommendations for a government aiming to ensure continuity of services and equitable distribution of vaccine. The strongest recommendation is for the government to aim to increase coverage, which will reduce inequality for all states and regions that were pro-rich. If donor transition means a reduction in the overall envelope, lessons can be learnt from regions such as Mandalay and Ayeyarwady to ensure that the poorest members of society are included in vaccination programmes rather than excluded, ensuring there is not a systematic pro-rich bias in EPI in future.

## Conclusions

Overall, the wealthier and more educated households used the publicly financed EPI more than poorer and less educated households in Myanmar. Considering the upcoming donor transition, national and regional governments should plan to maintain vaccine coverage while improving equity of vaccine services, especially for children of lower SES, mothers with less antenatal care visits and lower paternal education living in urban areas and conflict-affected remote regions. We hope this will help reduce under-five morbidity and mortality and outbreaks of vaccine-preventable disease, and also help achieve UHC in Myanmar. Future studies should address the weaknesses mentioned above and explore the reasons for heterogeneity of distribution of childhood immunisation across R&S.

10.1136/bmjgh-2021-007800.supp2 Supplementary data

## Data Availability

Data are available in a public, open access repository. All data we used for this work are publicly available. We provided information in the reference list.
