# Supplementary material for: Equity assessment of childhood immunisation at national and subnational levels in Myanmar: a benefit incidence analysis
Source: BMJ Glob Health. 2022 Jul 8;7(7):e007800. doi: 10.1136/bmjgh-2021-007800 (PMC9272074; doi:10.1136/bmjgh-2021-007800)
Supplement: Supplementary data [file bmjgh-2021-007800supp001.pdf]

Supplementary Appendix

Table S1. Background information of Regions and States, Myanmar

No	Regions & States	Per capita monthly income	Population Density (people per square km)	% of urban area
1	Yangon	94683	716	70
2	Mandalay	87892	200	35
3	Mon	86338	167	28
4	Tanintharyi	82865	32	24
5	Sagaing	67711	57	17
6	Naypyitaw	64629	164	32
7	Bago	61647	124	22
8	Magway	61216	87	15
9	Kayin	60076	52	22
10	Ayeyarwady	57665	177	14
11	Kachin	50337	19	36
12	Kayah	49493	24	25
13	Shan	45220	37	24
14	Rakhine	44926	87	17
15	Chin	31242	13	21

Table S2. Vaccine, syringe, and delivery costs by Regions and States, and urban and rural

Regions and States	BCG vaccine & injection supply cost	Delivery cost[1]	Total cost for BCG	Measles vaccine & injection supply cost	Delivery cost[1]	Total cost for Measles	DPT/ Pentavalent vaccine & injection supply cost	Delivery cost[1]	Total cost for DPT/ Pentavalent	OPV vaccine cost	Delivery cost[1]	Total cost for OPV	Full Immunization cost
**Hilly plateaus area**													
**Shan**													
Urban	0.37	2.23	**2.60**	0.94	2.23	**3.17**	2.90	6.69	**9.59**	0.39	6.69	**7.08**	**22.44**
Rural	0.37	2.22	**2.59**	0.94	2.22	**3.16**	2.90	6.66	**9.56**	0.39	6.66	**7.05**	**22.36**
**Delta area**													
**Ayeyarwady**													
Urban	0.37	1.44	**1.81**	0.94	1.44	**2.38**	2.90	4.32	**7.22**	0.39	4.32	**4.71**	**16.12**
Rural	0.37	2.79	**3.16**	0.94	2.79	**3.73**	2.90	8.37	**11.27**	0.39	8.37	**8.76**	**26.92**
**Central plain areas**													
**Mandalay, Yangon, Naypyitaw, Sagaing & Magway**													
Urban	0.37	1.79	**2.16**	0.94	1.79	**2.73**	2.90	5.37	**8.27**	0.39	5.37	**5.76**	**18.92**
Rural	0.37	1.79	**2.16**	0.94	1.79	**2.73**	2.90	5.37	**8.27**	0.39	5.37	**5.76**	**18.92**
**Coastal areas**													
**Tanintharyi, Rakhine & Mon**													
Urban	0.37	1.60	**1.97**	0.94	1.60	**2.54**	2.90	4.80	**7.70**	0.39	4.80	**5.19**	**17.40**
Rural	0.37	2.54	**2.91**	0.94	2.54	**3.48**	2.90	7.62	**10.52**	0.39	7.62	**8.01**	**24.92**
**Mountain range areas**													
**Kachin, Kayah, Kayin, Chin & Bago**													
Urban	0.37	1.48	**1.85**	0.94	1.48	**2.42**	2.90	4.44	**7.34**	0.39	4.44	**4.83**	**16.44**
Rural	0.37	1.92	**2.29**	0.94	1.92	**2.86**	2.90	5.76	**8.66**	0.39	5.76	**6.15**	**19.96**

1.Delivery cost includes labor (paid human resources), supply chain (cost for cold chain equipment, vehicles, transport, etc.) service delivery (cost for program management, training, etc.) and capital (buildings, utilities, other overheads, etc.)

Table S3. A list of variables and data sources

Variables	Data Sources
Childhood vaccination records	Vaccination card as primary source Mother's recall if primary source was missing (Myanmar Demographic and Health Survey)
Vaccine cost	"Costs of vaccinating a child" published by UNICEF
Syringe cost	"Costs of vaccinating a child" published by UNICEF
Delivery cost	Immunization Delivery Cost Catalogue
Geographical cost variation in Myanmar	"Assessing the operational costs of routine immunization activities at the sub-center level in Myanmar: What matters for increasing national immunization coverage?" by Aye et al.
Number of 2-year-old children	United Nations Population Division

Table S4. Percentage and number of sample population (12-23 months old children) living across five wealth quintiles by Regions and States, and urban and rural

Regions & States	Total Sample population	Poorest	Poorer	Middle	Richer	Richest
Nationwide	851.50	166.40	173.10	154.80	157.50	199.70
	100%	20%	20%	18%	18%	23%
Urban	220.28	8.87	14.25	18.60	41.76	136.80
	100%	4%	6%	8%	19%	62%
Rural	631.29	157.50	158.90	136.20	115.80	62.89
	100%	25%	25%	22%	18%	10%
Yangon	98.65	4.86	13.97	11.19	15.21	53.42
	100%	5%	14%	11%	15%	54%
Mandalay	88.55	5.00	8.43	14.47	21.48	39.17
	100%	6%	10%	16%	24%	44%
Mon	25.95	5.32	2.37	7.05	4.65	6.56
	100%	21%	9%	27%	18%	25%
Tanintharyi	22.36	4.07	5.43	3.25	5.07	4.54
	100%	18%	24%	15%	23%	20%
Sagaing	78.99	2.84	17.02	24.83	24.30	10.00
	100%	4%	22%	31%	31%	13%
Naypyitaw	17.93	2.85	6.01	3.00	2.51	3.56
	100%	16%	34%	17%	14%	20%
Bago	74.84	14.45	14.49	12.04	20.52	13.34
	100%	19%	19%	16%	27%	18%
Magway	55.23	14.88	7.21	9.74	12.79	10.61
	100%	27%	13%	18%	23%	19%
Kayin	28.21	5.89	6.50	5.48	5.56	4.78
	100%	21%	23%	19%	20%	17%
Ayeyarwady	125.20	52.55	39.11	21.09	3.11	9.34
	100%	42%	31%	17%	2%	7%
Kachin	25.79	1.92	5.38	5.42	8.81	4.26
	100%	7%	21%	21%	34%	17%
Kayah	5.60	0.75	1.01	1.60	1.35	0.89
	100%	13%	18%	29%	24%	16%
Shan	126.66	20.71	23.50	25.28	23.30	33.87
	100%	16%	19%	20%	18%	27%
Rakhine	66.48	26.70	21.05	7.23	6.97	4.53
	100%	40%	32%	11%	10%	7%
Chin	11.19	3.64	1.66	3.11	1.91	0.87
	100%	33%	15%	28%	17%	8%

Table S5. Basic vaccine coverage among children aged 12-23 months, by Regions and States, and urban and rural

Regions & States[1]	BCG	MCV	DPT/Pentavalent[2]	OPV[2]	Full Immunization[3]
**Nationwide**	87.8	77.1	62.3	67.0	54.8
**Urban**	91.84	81.69	75.22	76.04	67.47
**Rural**	86.39	75.45	57.79	63.91	50.40
Mandalay	93.4	86.5	88.2	90.2	81.3
Kayah	100.0	95.6	84.8	84.8	80.4
Yangon	96.4	79.7	76.0	78.0	67.4
Sagaing	86.5	76.9	71.5	71.5	66.4
Kayin	88.4	82.6	70.9	72.5	65.0
Mon	95.4	84.4	68.7	75.3	64.4
Kachin	91.2	81.9	73.6	70.4	59.4
Magway	97.8	91.0	61.8	68.2	58.2
Chin	92.7	73.0	64.7	69.9	53.0
Tanintharyi	98.1	85.0	61.8	67.3	52.4
Naypyitaw	97.7	85.8	59.9	59.6	49.4
Bago	94.5	77.6	56.3	59.0	46.7
Shan	76.1	63.7	53.9	52.7	45.8
Rakhine	88.1	73.4	48.4	72.3	41.0
Ayeyarwady	74.5	70.6	40.8	51.6	33.8

1. Regions and states ranked by full immunization coverage rate
2. Coverage rate for three doses of DPT/Pentavalent. Same for OPV
3. Full immunization is defined as completing the immunization schedule comprising of: BCG and measles and DPT1, DPT2, DPT3, OPV1, OPV2 and OPV3

Table S6. Immunization coverage according to wealth quintiles (WI)

Wealth Quintile	BCG	Measles	DPT/ Pentavalent	Polio	Full Immunization
Nationwide	87.80	77.07	62.30	67.04	54.82
Poorest	86.36	71.67	46.53	55.37	38.60
Poorer	80.15	71.22	52.07	57.81	44.02
Middle	81.94	68.77	57.15	60.32	51.42
Richer	91.15	81.17	69.57	74.98	61.37
Richest	97.51	89.83	82.58	83.73	75.17

Table S7. Immunization coverage according to maternal education (ME)

Maternal Education	BCG	Measles	DPT/ pentavalent	Polio	Full Immunization
Nationwide	87.8	77.1	62.3	67.0	54.8
No education	71.6	60.7	43.7	51.5	41.0
Primary	89.2	79.6	60.5	66.3	53.6
Secondary	91.0	77.1	68	69.2	56.4
Higher	96.0	91.3	83.7	90	79.6

Table S8. Decomposition of concentration index for full immunization utilization and benefits among children aged 12-23 months

Determinants	Full immunization Utilization				Full immunization benefits			
	Elasticity	Concentration Index	Contribution	Percent Contribution	Elasticity	Concentration Index	Contribution	Percent Contribution
**Wealth Quintiles (Ref: Poorest)**								
Poorer	0.027	-0.406	-0.011	-0.080	0.002	-0.406	-0.001	-0.007
Middle	0.087	-0.021	-0.002	-0.013	0.018	-0.021	0.000	-0.003
Richer	0.139	0.346	0.048	0.351	0.033	0.346	0.012	0.108
Richest	0.299	0.765	0.229	1.664	0.087	0.765	0.067	0.628
Sub total				**1.922**				**0.726**
**Urban/Rural (Ref: Rural)**								
Urban	0.026	0.481	0.012	**0.090**	-0.021	0.481	-0.010	**-0.094**
**Conflict Areas (Ref: Non-conflict area)**								
Currently Conflict area (Kachin, Rakhine and Shan)	-0.038	-0.067	0.002	0.018	0.009	-0.067	-0.001	-0.006
Conflict-affected areas (Kayah, Kayin, Chin and Tanintharyi)	0.030	-0.066	-0.002	-0.015	0.012	-0.066	-0.001	-0.008
Sub total				**0.004**				**-0.013**
**Mother's Education (Ref: No education)**								
Primary education	0.049	-0.180	-0.009	-0.065	0.020	-0.180	-0.004	-0.033
Secondary education	-0.068	0.244	-0.016	-0.120	-0.026	0.244	-0.006	-0.060
Higher education	0.021	0.660	0.014	0.102	0.000	0.660	0.000	0.000
Sub total				**-0.083**				**-0.094**
**Child's gender (Ref: Male)**								
Female	-0.160	-0.024	0.004	**0.028**	-0.051	-0.024	0.001	**0.012**
**Number of antenatal care (Ref: No)**								
< 4 times	0.113	-0.224	-0.025	-0.184	0.040	-0.224	-0.009	-0.084
>= 4 times	0.562	0.173	0.097	0.709	0.227	0.173	0.039	0.370
Sub total				**0.525**				**0.286**
**Place of delivery (Ref: Health care setting)**								
At home	0.078	-0.222	-0.017	**-0.125**	0.021	-0.222	-0.005	**-0.043**
**Father's Education (Ref: No education)**								
Primary education	0.020	-0.182	-0.004	-0.026	-0.006	-0.182	0.001	0.010
Secondary education	0.178	0.182	0.032	0.236	0.064	0.182	0.012	0.109
Higher education	0.012	0.666	0.008	0.060	0.004	0.666	0.002	0.023
Sub total				**0.271**				**0.141**
**Marital Status (Ref: Widowed, divorced, separated)**								
Married	0.321	0.003	0.001	**0.006**	0.101	0.003	0.000	**0.002**
**Mother's age (Ref: 15-24 years)**								
25-34 years	0.295	0.024	0.007	0.051	0.106	0.024	0.003	0.024
36-46 years	0.227	0.021	0.005	0.035	0.085	0.021	0.002	0.017
Sub total				**0.086**				**0.041**
**Tetanus immunization (Ref: No)**								
Yes	0.680	0.015	0.010	**0.073**	0.260	0.015	0.004	**0.0362**
Residual			-0.247				0.000	
Total concentration indices			0.137				0.106	

Table S9. Concentration indices of different types of immunizations by maternal education at urban and rural, and subnational levels (CI-ME-U and CI-ME-B)

	BCG	Measles	DPT	OPV	Full Immunization
**Urban**					
CI for Utilization	0.043	0.065	0.083	0.078	0.086
CI for Benefits	0.037	0.062	0.075	0.067	0.079
**Rural**					
CI for Utilization	0.025	0.020	0.050	0.036	0.031
CI for Benefits	0.015	0.011	0.043	0.026	0.022
**Mandalay**					
CI for Utilization	-0.008	-0.038	-0.014	-0.023	-0.046
CI for Benefits	-0.008	-0.038	-0.014	-0.023	-0.046
**Kayah**					
CI for Utilization	0.000	0.016	0.026	0.059	0.066
CI for Benefits	-0.010	0.008	0.021	0.053	0.061
**Yangon**					
CI for Utilization	0.005	0.031	0.048	0.033	0.064
CI for Benefits	0.005	0.031	0.048	0.033	0.064
**Sagaing**					
CI for Utilization	0.029	0.023	0.025	0.025	0.021
CI for Benefits	0.029	0.023	0.025	0.025	0.021
**Kayin**					
CI for Utilization	0.054	0.018	0.113	0.062	0.122
CI for Benefits	0.046	0.012	0.106	0.052	0.115
**Mon**					
CI for Utilization	-0.006	0.020	0.070	0.087	0.064
CI for Benefits	-0.042	-0.012	0.031	0.040	0.016
**Kachin**					
CI for Utilization	0.036	0.033	0.095	0.083	0.103
CI for Benefits	0.020	0.022	0.085	0.067	0.093
**Magway**					
CI for Utilization	0.007	-0.009	-0.051	-0.042	-0.036
CI for Benefits	0.007	-0.009	-0.051	-0.042	-0.036
**Chin**					
CI for Utilization	0.033	0.083	0.118	0.084	0.106
CI for Benefits	0.025	0.077	0.113	0.076	0.101
**Tanintharyi**					
CI for Utilization	0.007	-0.015	0.064	0.052	0.043
CI for Benefits	-0.010	-0.033	0.047	0.032	0.020
**Naypyitaw**					
CI for Utilization	0.009	0.030	0.107	0.139	0.208
CI for Benefits	0.009	0.030	0.107	0.139	0.208
**Bago**					
CI for Utilization	0.001	0.013	0.091	0.050	0.086
CI for Benefits	-0.013	0.003	0.074	0.028	0.068
**Shan**					
CI for Utilization	0.097	0.119	0.179	0.150	0.138
CI for Benefits	0.098	0.119	0.180	0.150	0.139
**Rakhine**					
CI for Utilization	0.020	0.084	0.145	0.029	0.159
CI for Benefits	0.004	0.064	0.127	0.000	0.136
**Ayeyarwady**					
CI for Utilization	-0.001	-0.018	-0.034	0.032	-0.076
CI for Benefits	-0.014	-0.035	-0.058	0.008	-0.099

Table S10. Concentration indices and achievement indices of full immunizations by wealth quintiles and maternal education at national and subnational levels

Regions & States	Immunization Coverage (µ)	Rank	By wealth quintiles CI-WI	Rank	AI-WI	Rank	By maternal education CI-ME	Rank	AI-ME	Rank
Nationwide	54.8		0.137		47.3		0.073		50.8	
Mandalay	81.3	1	-0.009	2	82.1	**1**	-0.047	2	85.1	**1**
Kayah	80.4	2	0.070	5	74.8	**2**	0.065	8	75.1	**2**
Yangon	67.4	3	0.115	9	59.6	**4**	0.064	6	63.0	**4**
Sagaing	66.4	4	0.018	3	65.2	**3**	0.021	4	65.0	**3**
Kayin	65.0	5	0.110	7	57.8	**5**	0.122	12	57.1	**7**
Mon	64.4	6	0.121	10	56.7	**6**	0.064	6	60.3	**5**
Kachin	59.4	7	0.121	10	52.3	**8**	0.103	10	53.3	**8**
Magway	58.2	8	0.060	4	54.7	**7**	-0.036	3	60.2	**6**
Chin	53.0	9	0.076	6	49.0	**9**	0.105	11	47.5	**10**
Tanintharyi	52.4	10	0.156	12	44.2	**10**	0.043	5	50.1	**9**
Naypyitaw	49.4	11	0.308	15	34.1	**14**	0.207	15	39.1	**13**
Bago	46.7	12	0.112	8	41.5	**11**	0.086	9	42.7	**11**
Shan	45.8	13	0.211	14	36.1	**13**	0.138	13	39.4	**12**
Rakhine	41.0	14	0.201	13	32.7	**15**	0.159	14	34.5	**15**
Ayeyarwady	33.8	15	-0.104	1	37.3	**12**	-0.076	1	36.4	**14**

AI-ME- Achievement indices by maternal education, AI-WI- Achievement indices by wealth quintiles, CI-ME- Concentration indices by maternal education, CI-WI- Concentration indices by wealth quintiles

Table S11. Extended concentration indices and achievement indices of basic immunization across wealth quintiles at national and subnational levels

Regions & States	Immunization Coverage (μ)	Rank	C (2)	Rank	I (2)	Rank	C (4)	Rank	I (4)	Rank	C (6)	Rank	I (6)	Rank
Nationwide	54.8		0.137		47.3		0.230		42.2		0.262		40.5	
Mandalay	81.3	1	-0.009	2	82.1	**1**	-0.047	2	85.2	**1**	-0.088	2	88.5	**1**
Kayah	80.4	2	0.070	5	74.8	**2**	0.169	7	66.8	**3**	0.239	8	61.1	**3**
Yangon	67.4	3	0.115	9	56.0	**4**	0.225	10	52.2	**5**	0.259	10	49.9	**5**
Sagaing	66.4	4	0.018	3	65.2	**3**	-0.013	3	67.3	**2**	-0.064	3	70.7	**2**
Kayin	65.0	5	0.110	7	57.8	**5**	0.147	5	55.4	**4**	0.131	4	56.5	**4**
Mon	64.4	6	0.121	10	56.7	**6**	0.262	12	47.5	**7**	0.338	13	42.6	**9**
Kachin	59.4	7	0.121	10	52.3	**8**	0.216	8	46.6	**8**	0.254	9	44.3	**8**
Magway	58.2	8	0.060	4	54.7	**7**	0.149	6	49.5	**6**	0.214	6	45.7	**6**
Chin	53.0	9	0.076	6	49.0	**9**	0.129	4	46.2	**9**	0.150	5	45.1	**7**
Tanintharyi	52.4	10	0.156	12	44.2	**10**	0.237	11	40.0	**10**	0.219	7	40.9	**10**
Naypyitaw	49.4	11	0.308	15	34.1	**14**	0.579	15	20.8	**15**	0.712	15	14.2	**15**
Bago	46.7	12	0.112	8	41.5	**11**	0.216	8	36.6	**12**	0.304	12	32.5	**12**
Shan	45.8	13	0.211	14	36.1	**13**	0.397	14	27.6	**14**	0.461	14	24.6	**14**
Rakhine	41.0	14	0.201	13	32.7	**15**	0.283	13	29.4	**13**	0.300	11	28.7	**13**
Ayeyarwady	33.8	15	-0.104	1	37.3	**12**	-0.167	1	39.4	**11**	-0.175	1	39.7	**11**

Table S12. Extended concentration indices and achievement indices of basic immunization across mother's education at national and subnational levels

Regions & States	Immunization Coverage (μ)	Rank	C (2)	Rank	I (2)	Rank	C (4)	Rank	I (4)	Rank	C (6)	Rank	I (6)	Rank
Nationwide	54.8		0.073		50.8		0.128		47.8		0.162		46.0	
Mandalay	81.3	1	-0.047	2	85.2	**1**	-0.077	3	87.6	**1**	-0.065	3	86.6	**1**
Kayah	80.4	2	0.065	8	75.1	**2**	0.146	9	68.6	**3**	0.191	10	65.0	**3**
Yangon	67.4	3	0.064	6	63.0	**4**	0.095	7	61.0	**5**	0.127	7	58.8	**5**
Sagaing	66.4	4	0.021	4	65.0	**3**	0.056	4	62.7	**4**	0.094	6	60.1	**4**
Kayin	65.0	5	0.122	12	57.1	**7**	0.267	14	47.7	**9**	0.350	14	42.2	**10**
Mon	64.4	6	0.064	6	60.3	**5**	0.116	8	56.9	**6**	0.153	8	54.6	**6**
Kachin	59.4	7	0.103	10	53.3	**8**	0.171	10	49.3	**7**	0.176	9	49.0	**7**
Magway	58.2	8	-0.036	3	60.2	**6**	-0.179	1	68.6	**2**	-0.295	1	75.3	**2**
Chin	53.0	9	0.105	11	47.5	**10**	0.209	11	42.0	**11**	0.259	12	39.3	**12**
Tanintharyi	52.4	10	0.043	5	50.1	**9**	0.065	5	49.0	**8**	0.068	5	48.8	**8**
Naypyitaw	49.4	11	0.207	15	39.1	**13**	0.363	15	31.4	**15**	0.429	14	28.2	**15**
Bago	46.7	12	0.086	9	42.7	**11**	0.068	6	43.5	**10**	0.031	4	45.3	**9**
Shan	45.8	13	0.138	13	39.4	**12**	0.266	13	33.6	**13**	0.311	13	31.5	**13**
Rakhine	41.0	14	0.159	14	34.5	**15**	0.224	12	31.8	**14**	0.244	11	31.0	**14**
Ayeyarwady	33.8	15	-0.076	1	36.4	**14**	-0.161	2	39.2	**12**	-0.227	2	41.5	**11**
