# Supplementary material for: Equity assessment of childhood immunisation at national and subnational levels in Myanmar: a benefit incidence analysis
Source: BMJ Glob Health. 2022 Jul 8;7(7):e007800. doi: 10.1136/bmjgh-2021-007800 (PMC9272074; doi:10.1136/bmjgh-2021-007800)
Supplement: Supplementary data [file bmjgh-2021-007800supp003.pdf]

A. National level immunization utilization and benefits across maternal education

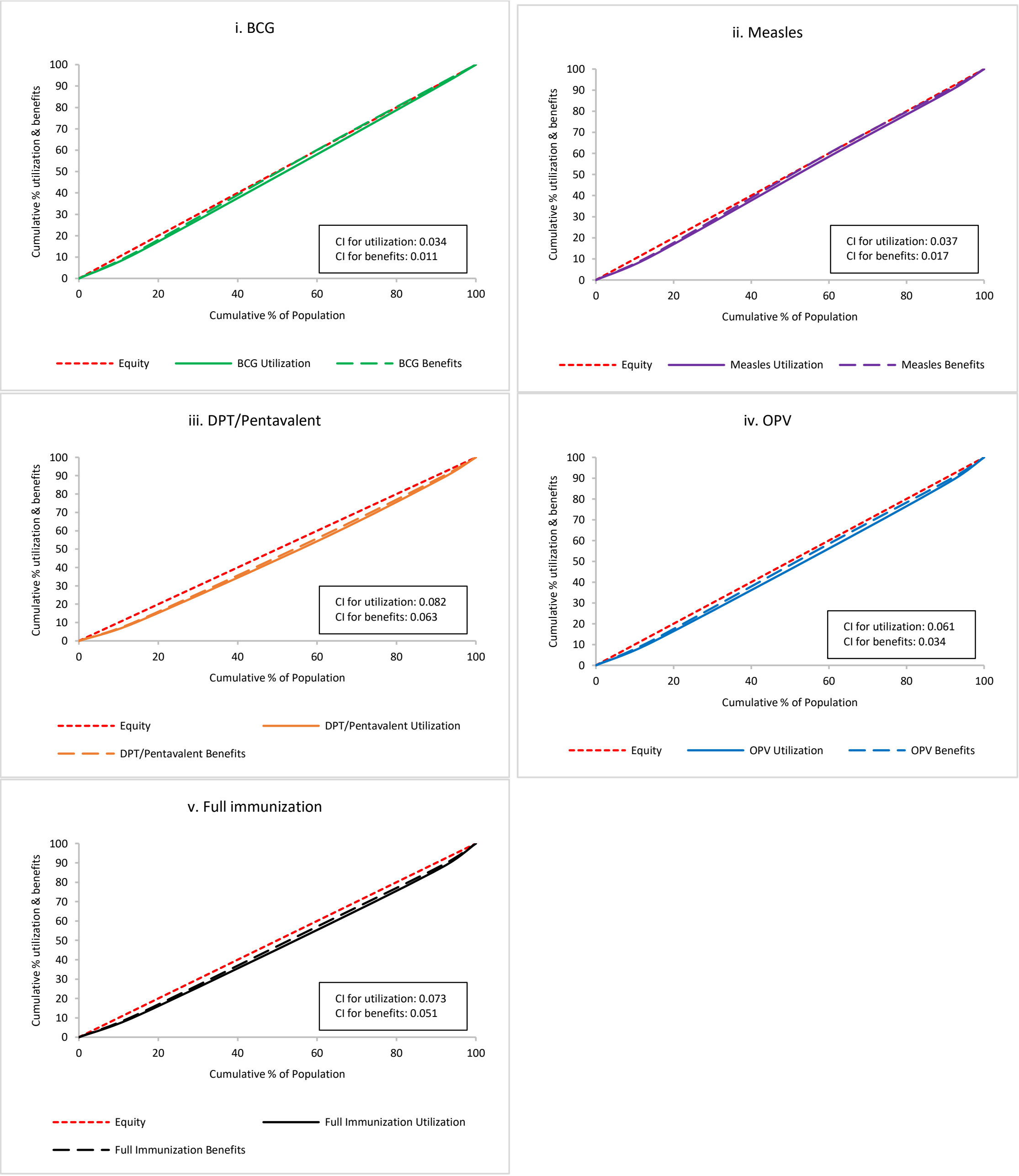

B. Comparison of urban/rural immunization benefits across maternal education

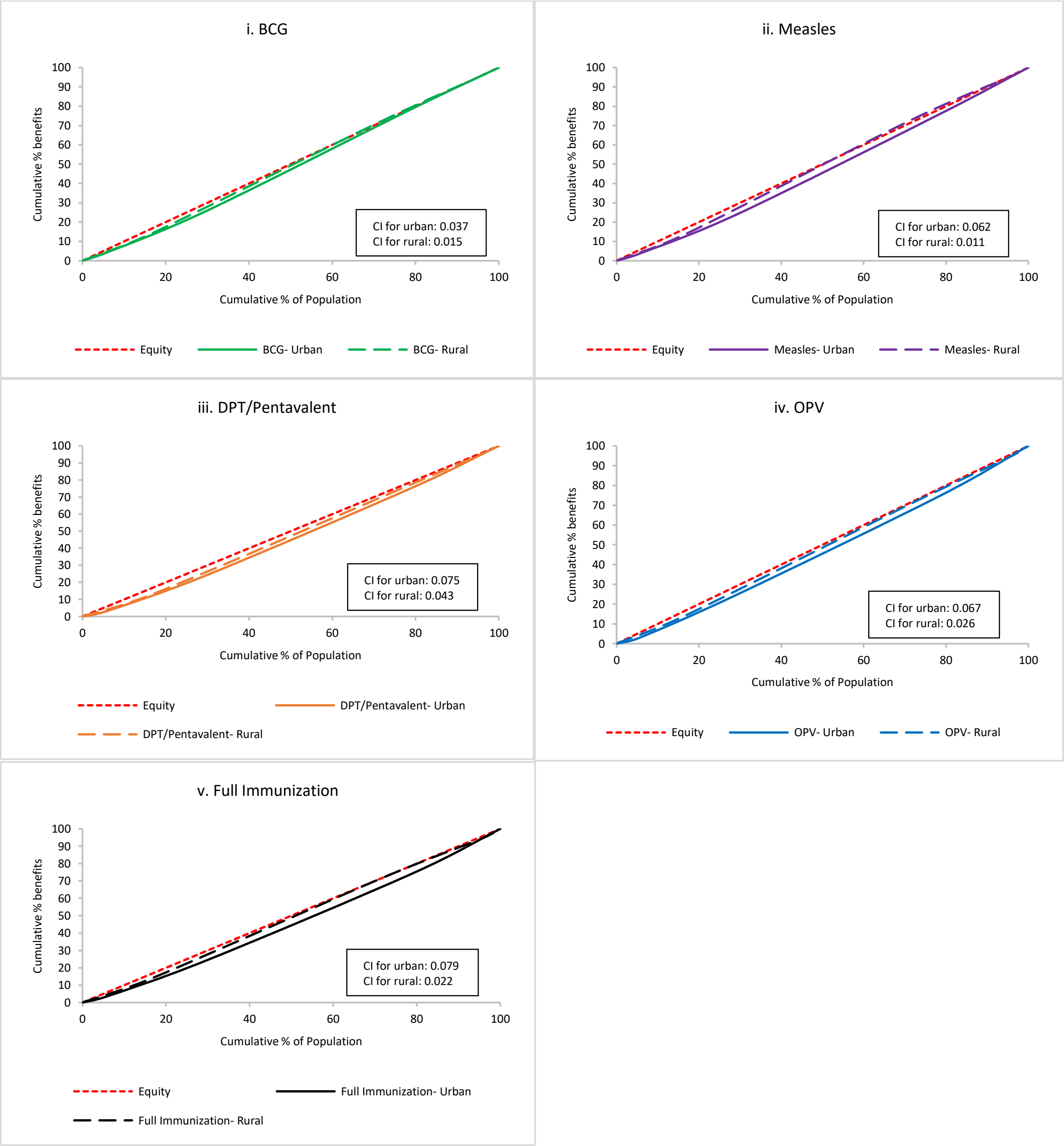

Figure S1.A National level immunization utilization and benefits across maternal education for i)BCG ii)Measles iii)DPT/Pentavalent iv)OPV and v)Full immunization
Figure S1.B Comparison of urban/rural immunization benefits across maternal education for i)BCG ii)Measles iii)DPT/Pentavalent iv)OPV and v)Full immunization
