# Supplementary material for: Equity assessment of childhood immunisation at national and subnational levels in Myanmar: a benefit incidence analysis
Source: BMJ Glob Health. 2022 Jul 8;7(7):e007800. doi: 10.1136/bmjgh-2021-007800 (PMC9272074; doi:10.1136/bmjgh-2021-007800)
Supplement: Supplementary data [file bmjgh-2021-007800supp002.pdf]

Appendix S1 – Reflexivity Statement

1. How does this study address local research and policy priorities?

Myanmar is expected to transition away from donor assistance. In other words, health services that are currently heavily funded by donors are at risk of losing funding. It is critical to understand which population groups benefit the most from donor funded health programs. This will provide helpful information for Myanmar government to plan for the health programs, after transition. With inputs from Myanmar co-authors (ZMW, TT, ZLK, KTH, PTC and TL), we conceptualized this study and conducted a benefit incidence analysis on the child immunization program.

2. How were local researchers involved in study design?

The first category of local researchers involved were those with extensive experience of involvement conducting, leading, or organising international research collaborations (ZMW, TT, ZLK, KTH, PTC and TL); they all work for a Myanmar-based NGO, Community Partners International (https://www.cpintl.org/) with extensive research experiences, particular the vulnerable communities, in Bangladesh, India, Myanmar (Burma), Nepal, Pakistan, Sri Lanka and Thailand.
The second category were local researchers with ongoing experience as health economists (ZMW, TT) and health services researchers (ZLK, KTH, PTC and TL) in Myanmar. In addition, there were high-income country researchers with extensive experience of conducting, leading, or organising international research collaborations involving low- and middle-income countries (TT, ASD, OO and WM). All authors have diverse cultural heritages originating from low- and middle-income countries.

3. How has funding been used to support the local research team?

This paper was part of a larger project funded by the Bill and Melinda Gates Foundation in six low- and middle-income countries. Specifically, the project has budgeted $25,000 to support the work in Myanmar. This has mainly been used to cover the time and efforts from the research team in Myanmar (travel budget has been cancelled due to COVID-19).

4. How are research staff who conducted data collection acknowledged?

We used publicly available secondary data and we have cited the data sources.

5. Do all members of the research partnership have access to study data?

ZMW is in charge of managing all data that has been used for this project. All member of the research partnership have access to the data.

6. How was data used to develop analytical skills within the partnership?

Team members were involved in all stages of analysis to leverage the multidisciplinary skills set of the partnership. We hosted biweekly meetings to discuss technical details of the project. OO, WM and TT led the overall design of the study with inputs from all authors. ZMW collected the data and performed the analysis with technical support from WM, ASD and TT. ZWM drafted the initial

manuscript and all coauthors contributed to the revision of the manuscript and approved the final version. All researchers gained experience of benefit incidence analysis.

How have research partners collaborated in interpreting study data?

Initial findings were shared and presented by ZMW with all. WM and OO provided technical interpretation and feedback on the findings, while TT, ZLK, KTH, PTC and TL interpreted the data focusing on the implications for Myanmar health system and health policy. All co-authors collaborated to agree on recommendations and contents of the manuscript.

7. How were research partners supported to develop writing skills?

WM, as a mid-career researcher, drafted outline of the manuscript. ZMW drafted the manuscript, reviewed by WM and TT for feedback. Later shared with all co-authors for comments and feedback. TT and OO, as senior researchers, provided final inputs on the advanced manuscript.

8. How will research products be shared to address local needs?

We will disseminate findings and recommendations to policymakers and public health researchers in Myanmar and other global organizations with research interests in Myanmar. For example, we organized a session at the iHEA meeting to disseminate initial findings (presented by ZMW). This paper will be published with public access. We also have plan to disseminate findings through policy dialogue in Myanmar.

9. How is the leadership, contribution and ownership of this work by LMIC researchers recognised within the authorship?

LMIC researchers were the leading authors of this paper ranked by their contribution to the paper. WM and OO conceptualize the study and provided technical support, and were the corresponding and last author.

10. **How have early career researchers across the partnership been included within the authorship team?**

All authors of this paper are early or mid-career researchers. ZMW, as early career researcher, is the first author of this paper, while OO and WM, as mid-career researchers, are last/corresponding authors.

11. How has gender balance been addressed within the authorship?

Six authors are female (ZMW, KTH, PTC, TL, ASD and WM) and three authors male (TT, ZLK and OO)

12. How has the project contributed to training of LMIC researchers?

The authorship team is primarily composed of early or mid-career researchers. TT, OO and WM are mid- careers, and have provided technical inputs on this paper. Early-career researchers were encouraged to take the lead with lots of hands-on explanation, training and technical inputs from mid-career researchers.

13. How has the project contributed to improvements in local infrastructure?

This project has not directly contributed to improvements in local infrastructure.

14. What safeguarding procedures were used to protect local study participants and researchers?

There was no primary data collection as part of this project, therefore this question is not directly applicable.
